# Fear effects on bank voles (Rodentia: Arvicolinae): testing for repellent candidates from predator volatiles

**DOI:** 10.1002/ps.6787

**Published:** 2022-01-26

**Authors:** Adrian Villalobos, Fredrik Schlyter, Göran Birgersson, Paweł Koteja, Magnus Löf

**Affiliations:** ^1^ Southern Swedish Forest Research Centre Swedish University of Agricultural Sciences Lomma Sweden; ^2^ Büsgen‐Institute, Department of Forest Zoology and Forest Conservation Georg‐August‐Universität Göttingen Göttingen Germany; ^3^ Department of Plant Protection Biology Swedish University of Agricultural Sciences Lomma Sweden; ^4^ Excellent Team for Mitigation, Faculty of Forestry & Wood Sciences Czech University of Life Sciences Prague Suchdol Czech Republic; ^5^ Institute of Environmental Sciences Jagiellonian University Kraków Poland

**Keywords:** rodent pest management, repellents, area avoidance, foraging, bank vole

## Abstract

**BACKGROUND:**

Arvicolinae rodents are known pests causing damage to both agricultural and forest crops. Today, rodenticides for rodent control are widely discouraged owing to their negative effects on the environment. Rodents are the main prey for several predators, and their complex olfactory system allows them to identify risks of predation. Therefore, the potential use of predators' scents as repellents has gained interest as an ecologically based rodent control method. In a two‐choice experiment, we investigated the potential repellent effects of five synthetic predator compounds: 2‐phenylethylamine (2‐PEA), 2‐propylthietane (2‐PT), indole, heptanal and 2,5‐dihydro‐2,4,5‐trimethylthiazoline (TMT), at 1% and 5% doses, using the bank vole (*Myodes glareolus*) as a rodent model.

**RESULTS:**

The compound 2‐PEA reduced both the food contacts and the time spent by voles in the treatment arm compared to the control arm. Likewise, 2‐PT‐treated arms reduced the food contacts, and the voles spent less time there, although this latter difference was not significant. Indole also showed a tendency to reduce the time spent at the treatment arm; however, this result was not significant. Unexpectedly, TMT had the reverse effect in showing attractive properties, possibly due to odor cues from differently sized predators and intraguild predation in nature. We found no dose‐related effects for any compounds tested.

**CONCLUSION:**

Our results suggest that the 2‐PEA and 2‐PT are both effective odor stimuli for triggering reduced food contacts and area avoidance, and they may be good repellent candidates. We suggest further testing of 2‐PEA and 2‐PT in field experiments to further determine their dose‐efficiency as repellents against rodents in more natural environments. © 2022 The Authors. *Pest Management Science* published by John Wiley & Sons Ltd on behalf of Society of Chemical Industry.

## Graphical abstract

In a two‐choice laboratory experiment, the chemical compounds 2‐phenylethylamine and 2‐propylthietane were the most effective in triggering avoidance‐related behaviors in bank voles, regardless of their dose. Indole showed modest results, whereas heptanal and 2,5‐dihydro‐2,4,5‐trimethylthiazoline (TMT) did not show the anticipated defense‐related effects.

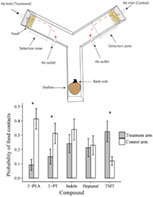

## 1 INTRODUCTION

Ground‐dwelling rodents of the subfamily Arvicolinae are among the major pests in both agricultural and forest crops in temperate and boreal ecoregions.^1^, ^2^ For example, damage to agricultural crops during Arvicoline rodent outbreaks has been reported to cause annual economic losses up to €700 million in Germany^1^ and up to 16% loss of farmers' income in Poland.^3^ In forestry, damage caused by rodents such as the bank vole (*Myodes glareolus* Schreber, 1780) also can be pronounced.^1^ For example, the early stages of forest regeneration often are damaged when rodents gnaw bark or clip young seedlings, causing seedling mortality.^4^ Moreover, bank voles also readily eat seeds such as acorns and beechnuts,^5^ which can compromise the outcome of direct seeding during forest restoration with broadleaved tree species.^6^

A common management strategy involves usage of anticoagulant rodenticides to reduce rodent populations.^7^ The use of this method, however, has been disallowed with the introduction of new regulations, especially among European countries.^1^ The reason for this is that the application of rodenticides represents a risk to the environment resulting from primary and secondary poisoning of nontarget species.^8^

Alternative strategies to rodenticides in forestry include, for example, (i) mechanical site preparation such as mounding, ploughing or scarification,^6^ (ii) the reduction of ground vegetation,^9^, ^10^ (iii) physical barriers^11^ and (iv) the use of supplementary food.^12^ These methods, however, may increase the costs and time required for management operations and still could have negative effects on the environment and crop production. One method which could reduce both environmental impacts and management costs is the use of plant secondary metabolites^13^ and predators' scents to deter rodents from seeds,^14^, ^15^ but little research has been conducted in this subject.

The rodent's complex olfactory system plays a key role in foraging,^16^ identifying conspecifics^17^ and detecting the presence of predators.^18^ As the main prey for several mammalian predators, rodents constantly experience a trade‐off between foraging effort and risk of predation.^18^, ^19^ This is the basis of the ‘predation risk allocation hypothesis”, which predicts a low foraging effort during short periods of high predation risk;^20^ in other words, animals need to fulfill their dietary and bodily needs without being preyed upon. Therefore, one would expect defense behaviors to be active only when the prey animal can accurately identify a high predation risk.^21^ Mammalian predators produce scent marks or odors for intraspecific communication such as individual recognition, breeding and territory marking.^22^ However, these odors also may act as kairomones triggering fear or defense responses in prey species.^23^ In the presence of predator odors, rodents use detection avoidance, shift their movements to safe habitats, and decrease foraging or feeding as primary defense mechanisms.^24^, ^25^ Hence, there is great interest in the application of predator odors as repellents in an ecologically‐based rodent management framework to replace rodenticides.^15^, ^19^

Several volatile compounds have been identified from the odor of feces, urine and anal glands of mustelids,^26^, ^27^, ^28^ and feces of the red fox (*Vulpes vulpes* L., 1758).^29^ Both are important predators of rodents in northern and central Europe.^30^, ^31^ Some of these compounds have been widely studied as fear‐inducing odors in rodents.^18^, ^32^ For example, the mustelid compound 2‐propylthietane (2‐PT) elicited avoidance in rodents during application as a single compound in laboratory studies^33^, ^34^, ^35^, ^36^ or reduced rodent damage to seedlings during field application in a 1:1 mixture with 3‐propyl‐1,2‐dithiolane.^15^, ^37^ The compound indole is derived from anal glands of different mustelids. In a 16:1:4 mixture with 2‐PT and 3‐propyl‐l,2‐dithiolane, it induced fear effects by suppressing feeding behavior in meadow voles (*Microtus pennsylvanicus* Ord, 1815) and montane voles (*Microtus montanus* Peale, 1848).^14^ However, the application of indole as a single compound has not been studied. Another compound that elicits avoidance behavior in rodents is the biogenic amine 2‐phenylethylamine which has been found in mustelids and other mammalian carnivores.^27^

Of all predator odor compounds, TMT (2,5‐dihydro‐2,4,5‐trimethylthiazole) is the most studied in laboratory rodents.^38^, ^39^ It was first identified as the most active chemical constituent of fox feces odor, inducing different fear‐related responses in rats.^29^ Since then, there has been substantial evidence for fear‐related behaviors in rodents during TMT exposure in laboratory studies.^40^, ^41^ However, there also are contrary evidence where the expected fear response to TMT could not be observed (reviewed in Fendt and Endres,^40^ and Apfelbach *et al*.^18^).

Aside from predator odors derived from feces, urine or anal glands, fur/skin‐derived odors may trigger stronger avoidance responses and longer‐lasting effects, as they could be a more reliable olfactory indicator of predators' presence.^42^ However, there is no current information regarding active compounds present in predators' fur/skin, which can produce repellent effects.^18^

Above all, there is conflicting knowledge regarding whether single compounds or complex blends (‘bouquets’) of compounds better elicit fear responses in rodents.^36^, ^43^, ^44^ In a recent review, Apfelbach *et al*.^43^ concluded that single compounds present little information about the presence of a predator, and only complex arrays of compounds can elicit reliable information. However, there also is evidence that responses to some single compounds are as strong as more complex mixtures.^44^, ^45^, ^46^ Moreover, Jackson *et al*.^44^ underlines that animal response to a single compound is more dose‐dependent. Indeed, Apfelbach *et al*.^18^ suggests that intermediate doses may give stronger repellent effects depending on the target species.

As a step toward using predator scent in rodent pest management, we investigated the potential repellent effects on bank voles of four synthetic odors of different predators (red fox and mustelids) and one volatile compound found in mink fur (*Neovison vison* Schreber, 1777) in a two‐choice laboratory experiment. The specific objectives of this study were to: (i) compare the potential repellent efficacy of the selected synthetic predator scent compounds, (ii) compare these compounds at two different doses, and (iii) assess the performance of the compounds in promoting two different behaviors: food contact reduction and area avoidance.

## 2 MATERIALS AND METHODS

### 2.1 Animals

In total, 33 female and 33 male laboratory‐bred bank voles (*M. glareolus*) were chosen randomly from unselected control lines (26th generation) of an ongoing artificial selection experiment.^47^ Bank voles weighed 20–30 g and were between two and five months old. The animals were kept in same‐sex groups of two or three individuals in standard rodent plastic cages (Tecniplast, Buguggiatte, Italy; dimensions L × W × H: 267 × 207 × 140 mm; floor area 370 cm^2^) with sawdust bedding. The temperature (20 °C ± 1 °C) and photoperiod (16 h:8 h, light:dark) remained constant.

### 2.2 Chemical compounds

We used single synthetic volatile compounds (compound treatment) representing predator odor cues previously described from Mustelidae or Canidae (Table 1). The chemical compounds tested were: (i) 2‐phenylethylamine (2‐PEA, ≥99% purity; Sigma‐Aldrich, Darmstadt, Germany), a general component of carnivores' smell, (ii) 2‐propylthietane (2‐PT, ≥95% purity; Chemspace, Riga, Latvia) from anal gland secretions of mustelids, (iii) indole (≥99% purity; Sigma Aldrich) from anal gland secretions of mustelids, (iv) heptanal (≥95% purity; Sigma Aldrich) identified as the most pronounced compound from mink fur (Supporting information, Table S1), and (v) 2,5‐dihydro‐2,4,5‐trimethylthiazoline (2,5‐TMT, ≥97%; Bio SRQ, Sarasota, FL, USA) from fox feces. Each compound was diluted, using the solvent pentane, to two different doses (1% w/w and 5% w/w). The compound heptanal was obtained by screening volatile organic compounds from ten samples of mink fur with the use of dynamic headspace sampling and analyzed with a gas chromatograph coupled with a mass spectrometer [see Methods for headspace collection of mink fur (Appendix S1)].

**Table 1:** List of synthetic predator odors tested as rodent repellents in the experiment. Names of predators denote the animal reference from which the compound was identified. If the compound is described from several animal species, their order or family is given

Chemical compound	Predator	CAS no.	Molecular weight (g mol^–1^)	Source/Reference	Vendor
2‐Phenylethylamine (2‐PEA)	Carnivora	64‐04‐0	121.18	Ferrero *et al*.^27^	Sigma‐Aldrich (Germany)
2‐Propylthietane (2‐PT)	Mustelidae	70678‐49‐8	108.14	Crump^77^	Chem‐Space (Latvia)
Indole	Mustelidae	120‐72‐9	117.15	Brinck *et al*.^78^	Sigma‐Aldrich (Germany)
Heptanal	Mink	111‐71‐7	114.19	Supplementary material, Appendix S1	Sigma‐Aldrich (Germany)
2,5‐Dihydro‐2,4,5‐trimethylthiazoline (TMT)	Red fox	60633‐24‐1	129.22	Vernet‐Maury^29^	SrqBio Inc. (USA)

### 2.3 Y‐maze design

**Figure 1:**
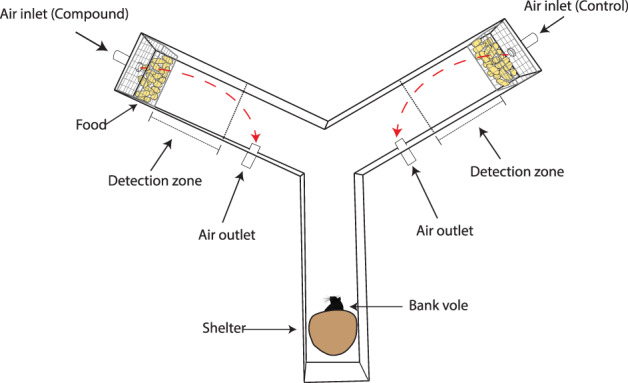
Design of the Y‐maze used in the behavioral experiments. Food (rodent chow) was placed in small mesh cages (mesh size 12.5 mm) in the treatment and control arms. The detection zone used in the behavior analysis is marked with perpendicular lines in the maze at 10 cm from the food source. At the sides of the maze, an outlet connected to a reversed air pump pulls air out of the maze. Dashed arrows show the odor plume directions. A coconut half‐shell was used as a shelter for the animal in the stem arm of the maze.

Animals were tested in a two‐choice design using three Y‐shaped mazes, modified after Engman *et al*.^48^ Each Y‐maze consisted of three aluminum arms (attached by the arm walls at 120° angles) each measuring 37.5 × 15 × 11 cm (Fig. 1). The ceiling cover was made of transparent acrylic glass and the ground base of polyamide plastic. The stem (lower, straight arm) was provided as a ‘shelter’ zone with a coconut half‐shell placed as a shelter at the base. Odors were distributed with flexible polytetrafluoroethylene (PTFE) tubing to a centered air inlet including a 5 × 2 cm polyamide cylinder in each of the two ‘treatment’ arm ends. To avoid the odor plumes entering the shelter zone of the maze, two air outlets at 25 cm from the arm ends were connected with PTFE tubing to a reversed air pump (Fig. 1).

### 2.4 Experimental design

The experiment was carried out between August and October 2018 at the Institute of Environmental Sciences (Jagiellonian University, Krakow, Poland). In the Y‐mazes, the air inlets were connected to an odor source, which consisted of a sealed 2‐mL vial with a PTFE tube (length 40 mm; inside diameter 3 mm) filled with cotton yarn wick inserted into a hole drilled in its screw cap (‘wick‐baits’^49^). Inside the vial, the yarn was soaked with 500 μL of the compound treatment. The ‘wick‐bait’ was placed inside a washing bottle connected to the PTFE tubing (inside diameter 1.5 mm). In order to reduce fatigue due to odor saturation, the odor was first pumped into the maze for 1 min after the first 5 min of the trial, and thereafter every 5 min for 1 h (ten air puffs of 1 min each in total). Odors were pumped into the maze by a diaphragm vacuum pump (KNF Neuberger, Freiburg, Germany) at a rate of 800 mL min^–1^. Pumped air was filtered with activated charcoal to reduce air impurities before being mixed with the odor. Which Y‐maze arm (left or right) the odor/treatment was pumped into was selected randomly, and clean air was pumped into the untreated arm as the control. In both the control and the treatment arms, the air was pumped out of the maze at 1500 mL min^–1^. At each end of both odor arms, standard rodent chow (Labofeed H, Kcynia, Poland) was placed in small mesh cages (mesh size 12.5 mm) as a food source (cf. Fig. 1). The food cages allowed voles to gnaw on the food pellets but without removing them from the cage. For each trial three Y‐mazes were allocated in the experimental room. To avoid cross‐reactions, the three Y‐mazes were placed 100 cm away from each other. After the end of each trial, all materials were washed thoroughly with odorless soap, and 95% ethanol and Y‐mazes were replaced by three clean ones.

The animals had not experienced any other experimental manipulation since birth and were subjected to the experimental procedure only once. For each compound and dose combination, six naive bank voles were tested. To reduce stress resulting from handling or reaction to novel environments, the animals were acclimatized to the Y‐maze environment for ≥10 h (light photoperiod). Trials started during the first hour of the artificial night cycle and after acclimatization to the experimental setup. Neither the animals nor the Y‐mazes were manipulated during and between acclimatization and the trials. The full 60 min of each trial was video‐recorded (resolution 704 × 576 pixels, 25 fps). Because trials started at the beginning of the night cycle, red LED lights (>80 Ra) were mounted beneath the mazes to enhance the contrast of the animals in the video recordings. Water and food (rodent chow; Labofeed H) were supplied *ad libitum* during acclimatization, and between and during the trials.

All trials were done in compliance with Polish animal welfare laws (ethical permit no. 258/2017; 2nd Local Institutional Animal Care and Use Committee; Institute of Pharmacology, Polish Academy of Sciences; Krakow, Poland).

### 2.5 Measurements

For each of the ten air puffs described above, we recorded two different behaviors: number of food contacts and area avoidance. To determine food contact (as an approximation of foraging), we recorded whether an animal poked its nose into the food cage (Fig. 1) for >5 s consecutively during each 1‐min air puff, and food contact was noted as 1 or as 0 (no contact). To measure area avoidance, we defined a 10‐cm virtual detection zone from the food cage to the center of the Y‐maze at each choice arm (Fig. 1) and measured the cumulative time spent in each detection zone only during each 1‐min air puff. Video analyses were carried out with the video tracking software EthoVision XT 11 (Noldus Information Technology, Wageningen, The Netherlands; license no. EV120‐06434‐AAJIIAAA‐R).

### 2.6 Statistical analysis

R v3.5.0^50^ was used for all analyses. During the experiment, one female of the 1% TMT treatment did not leave the shelter and was therefore removed from the dataset. Food contact (binary response variable) was analyzed using a generalized linear mixed model (GLMM) with a binomial error distribution and a logit link in R/lme4.^51^ We included the following factors and their interactions as fixed effects: *compound* (five levels) × *dose* (two levels) × *Y‐maze arm type* (treatment/control arm, two levels). Random factors were included as *vole* crossed with *air puff* to account for repeated measures (ten air puffs).

Area avoidance was analyzed using a zero‐inflated GLMM with R/glmmTMB.^52^ Here we included cumulative time spent in the detection zone as the response variable. To account for the semi‐continuous nature of the response variable (a significant number of zeros combined with a positive continuous distribution), we specified a Tweedie error distribution.^53^ The same fixed and random factors as in the food contact model were used.

In order to evaluate the factor effects and their interactions for both the food contact and area avoidance models, an analysis of deviance for unbalanced design (Wald *X* ^2^ Type III) was performed. If significant interactions were observed, *post hoc* tests for multiple comparisons between factor levels were implemented using estimated marginal means with R/emmeans.^54^ All models were examined for overdispersion and residual distribution using the functions ‘testDispersion’ and ‘testUniformity’ from R/DHARMa.^55^ The α‐level for all statistical tests was set at 0.05.

## 3 RESULTS

### 3.1 Food contact

**Figure 2:**
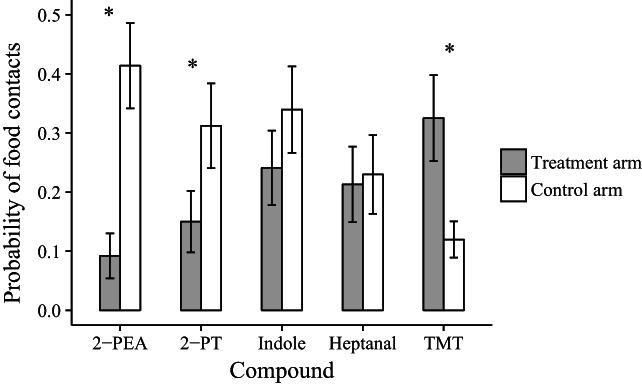
Probability of food contacts (nose pokes into food cage for >5 s) during ten air puffs for each compound treatment. As dose (1% and 5%) and its interactions were not significant, these results are averaged (*n* = 12 animals) for each compound. Asterisks indicate significant differences between the arms of the Y‐maze. Error bars show the standard error of the mean probability of nose pokes per treatment.

Y‐maze arm type (treatment *versus* control) strongly affected the probability of food contact by bank voles, but the direction and magnitude of the effect depended on the compound used (Table 2). The compounds 2‐PEA and 2‐PT significantly reduced the probability of food contact in the treatment arm compared to the control arm (Fig. 2; Table S2). Likewise, although nonsignificant, indole decreased food contact (Fig. 2). Food contacts were not affected by the presence of heptanal (Fig. 2; Table S2). Surprisingly, the presence of TMT, the odor derived from red fox feces, resulted in an increase, rather than the expected decrease in food contact in the treatment arm (Fig. 2; Table S2). This increase of food contact with TMT was significantly different compared to the probability of food contacts with 2‐PEA and 2‐PT in the treatment arm (Table 3). However, no significant differences between the two dose levels (1% and 5%) were observed for direct effects or interactions with other factors (Table 2).

**Table 2:** Fixed factors and their interactions' predictive power of food contacts by bank voles. Interactions were derived *a posteriori* from a generalized linear mixed model with a binomial error distribution using an analysis of deviance (Wald *X* ^2^ Type III). For description of fixed factors, see text

Fixed factors and interactions	*χ* ^2^	df	*P* (>| *χ* ^2^|)
Compound	12.38	4	0.01
Dose	0.77	1	0.38
Y‐maze arm type	14.37	1	<0.01
Compound × Dose	6.26	4	0.18
Compound × Y‐maze arm type	30.33	4	<0.01
Dose × Y‐maze arm type	0.18	1	0.67
Compound × Y‐maze arm type × Dose	6.83	4	0.16

**Table 3:** Pairwise comparisons among the five compounds' relative probabilities of food contacts in the treatment and control arms during ten odor puffs. Results were derived *a posteriori* from a generalized linear mixed model with a binomial error distribution using an interactions analysis with estimated marginal means (EMMEANS). Estimate values are back‐transformed to the response variable (probability of food contact)

Compound	Estimate	SE	*z‐*ratio	*P* (>|*z*|)
Compound arm				
2‐PEA – 2‐PT	−0.05	0.05	−0.95	0.88
2‐PEA – Indole	−0.14	0.07	−1.98	0.28
2‐PEA – Heptanal	−0.10	0.06	−1.63	0.48
2‐PEA – TMT	−0.22	0.08	−2.63	0.01
2‐PT – Indole	−0.09	0.07	−1.25	0.72
2‐PT – Heptanal	−0.06	0.07	−0.84	0.92
2‐PT – TMT	−0.18	0.09	−2.02	0.03
Indole – TMT	−0.08	0.09	−0.84	0.92
Heptanal – Indole	−0.04	0.08	−0.42	0.99
Heptanal – TMT	−0.12	0.09	−1.25	0.72
Control arm				
2‐PEA – 2‐PT	0.12	0.11	1.16	0.78
2‐PEA – Indole	0.09	0.11	0.80	0.93
2‐PEA – Heptanal	0.21	0.10	2.08	0.23
2‐PEA – TMT	0.30	0.09	3.29	<0.01
2‐PT – Indole	−0.03	0.10	−0.33	1.00
2‐PT – Heptanal	0.08	0.09	0.93	0.89
2‐PT – TMT	0.19	0.08	2.49	0.09
Indole – TMT	0.22	0.07	2.78	0.04
Heptanal – Indole	−0.12	0.10	−1.23	0.74
Heptanal – TMT	0.09	0.07	1.37	0.64

### 3.2 Area avoidance

**Figure 3:**
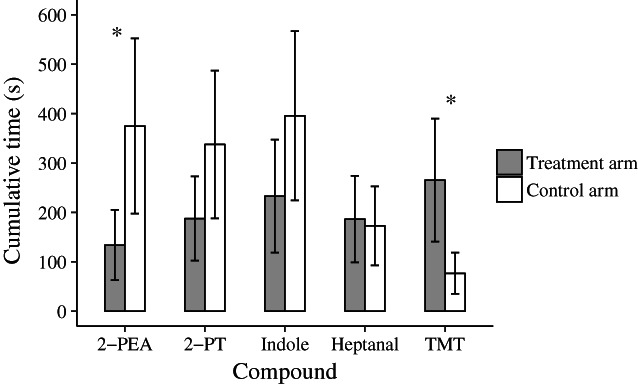
Cumulative time spent by bank voles in the detection zone of the treatment arm (gray bars) and control arm (white bars) during ten air puffs for all compound treatments. As dose (1% and 5%) and its interactions were not significant, these results are averaged (*n* = 12 animals) for each compound. Asterisks indicate significant differences between the arms of the Y‐maze. Error bars show the standard error of the mean cumulative time spent by bank voles.

The results from measurements of area avoidance were consistent with the results of the food contact. Y‐maze arm type and its interaction with the compound showed strong effects on the cumulative time spent by bank voles in the detection zones (Table 4). In the presence of 2‐PEA the cumulative time spent by bank voles in the treatment arm was significantly reduced by ≈240 seconds (s) compared to the control arm (Fig. 3; Table S3). Likewise, although not significantly, the mean cumulative time spent in the treatment arm in the presence of the compounds 2‐PT and indole was (respectively) 150 s and 160 s less than time in the control arm (Fig. 3; Table S3). Bank voles did not avoid the compounds heptanal or TMT (Fig. 3). Surprisingly, but again consistent with results of the food contact analysis, the rodents were attracted to TMT by a cumulative time in the treatment arm lasting 190 s more than in the control arm (Fig. 3, Table S3). Moreover, dose and its interactions were not significant in our model (Table 4).

**Table 4:** Fixed factors and their interactions on time spent in the detection zones by bank voles. Interactions were derived *a posteriori* from a generalized linear mixed model with a Tweedie error distribution using an analysis of deviance (Wald *X* ^2^ Type III). For a description of the fixed factors see text

Fixed factors and interactions	*χ* ^2^	df	*P* (>| *χ* ^2^|)
Compound	5.79	4	0.22
Dose	3.11	1	0.08
Y‐maze arm type	6.85	1	<0.01
Compound × Dose	4.93	4	0.30
Compound × Y‐maze arm type	19.65	4	<0.01
Dose × Y‐maze arm type	0.52	1	0.42
Compound × Y‐maze arm type × Dose	5.19	4	0.27

## 4 DISCUSSION

In our study, the more general carnivore compound 2‐PEA was the most active repellent candidate, followed by 2‐PT and modestly by indole. The mink fur compound heptanal did not show any avoidance effects. However, the fox feces compound TMT instead had attractive activity. Thus, our results suggest that regardless of the doses tested, the presence of some tested compounds could increase or decrease the probability of food contact. These results were relatively consistent whether analyzed in terms of food contact or time spent in zones with odors.

Different field and laboratory studies have shown that predators' odors indeed produce distinctive defense behaviors such as decreased foraging or feeding and area avoidance.^18^, ^24^ Therefore, we selected reduced food contact as an approximation of foraging and time spent in the detection zones as a proxy for area avoidance.

There is, to our knowledge, no current information about foraging reduction or feeding suppression effects in rodents by the compound 2‐PEA. Thus, our results suggest, for the first time, such behavioral effects in rodents. Several other avoidance effects have been observed in mice and rats during exposure to 2‐PEA.^27^, ^56^ For example, and in line with our results, Ferrero *et al*.^27^ found that 2‐PEA elicits innate avoidance behavior in mice and rats and increases plasma corticosterone levels, indicating stress. Furthermore, those authors showed that 2‐PEA is a general component present in different carnivore odors, and subsequently it was identified as a predator odor‐derived kairomone. In another study by Wernecke,^56^ the avoidance response to 2‐PEA in rats was observed only at low doses.

The feeding inhibition patterns observed in our results when the compound 2‐PT was applied are consistent with previous laboratory experiments from Heale and Vanderwolf.^57^ They observed a reduced consumption of rodent food pellets by Long–Evans rats (*Rattus norvegicus domestica* Berkenhout, 1769) when 2‐PT was applied close to the food. Moreover, Woolhouse and Morgan^58^ found that foliage of Monterrey pine (*Pinus radiata* D. Don) seedlings sprayed with 2‐PT were consumed less by common brushtail possums (*Trichosurus vulpecula* Kerr, 1792). Further field and laboratory studies using a 1:1 mixture of 2‐PT with 3‐propyl‐1,2‐dithiolane showed a reduction of bark and vascular tissue feeding on apple trees by meadow voles,^14^ less feeding on Scots pine seedlings by the southern red‐backed vole (*Myodes gapperi* Vigors, 1830)^37^ and less food retrieval by mountain beavers (*Aplodontia rufa* Rafinesque, 1817).^59^ Several laboratory studies also have found that 2‐PT‐treated areas are avoided by mice.^33^, ^34^, ^35^, ^36^ There are indications that mice are especially sensitive to 2‐PT, as Sarrafchi *et al*.^35^ found compound recognition by mice at low doses. From our experiments, we can only suggest that, regardless of its dose, 2‐PT may elicit avoidance responses in bank voles. However, the olfactory threshold of bank voles for 2‐PT or other predator odor compounds remains unknown. Clearly, the actual amount of a chemical reaching an animal's olfactory organs after release from a dispenser will differ considerably as a consequence of volatility, dependent on molecular mass and other chemical properties.^60^

In the presence of indole, the number of food contacts was not significantly different between the treatment and control arms. However, we found a nonsignificant tendency for area avoidance. To the best of our knowledge, indole has never been tested previously as a single compound in rodents but rather as a mixture with sulfurous^14^ or other nitrogenous^61^ compounds. These mixtures showed inconsistent results; although Sullivan *et al*.^14^ found feeding suppression in meadow voles and montane voles, Swihart *et al*.^61^ did not observe feeding reduction in meadow voles. Interestingly, indole combined with phenol deterred African elephants (*Loxodonta africana* Blumenbach, 1797), a much larger herbivore, from crossing a path, similar to its predators' feces odors in the field.^62^ Further laboratory and field experiments are needed to determine if indole as a single compound can be effective as a repellent against bank voles.

The compound heptanal, derived from mink fur, previously has been identified from other carnivorous mammals such as ferret (*Mustela furo* L., 1758) urine^28^ and otter (*Lutra lutra* L., 1758) spraint.^63^ However, our results did not show the expected avoidance effects. These results might be explained by heptanal also having been described as a more general odor compound found in products of nonpredator mammals such as cattle fur,^64^ and other organic materials such as flowers^65^ and soft cheese.^66^ Aside from our experiment, there are no other studies related to feeding inhibition or avoidance due to heptanal in rodents.

We did not observe the expected feeding inhibition and avoidance behavior with the compound TMT; surprisingly, bank voles were attracted to this compound. These results contrast with a previous study by Endres and Fendt^67^ on several rat strains, which reduced their feeding behavior when exposed to TMT. Similarly, Burwash *et al*.^68^ observed inhibitory behavior trends for feeding by wild roof rats (*Rattus rattus* L., 1758) in the presence of TMT in a two‐choice laboratory assay. Moreover, recent studies by Adduci *et al*.^69^ on the house mouse (*Mus musculus* L., 1758) and the brown rat (*Rattus norvegicus* Berkenhout, 1769) indicate only avoidance behavior in rats. Further studies described different avoidance responses depending on the rat strain.^40^, ^45^, ^70^, ^71^ For example, avoidance behavior was less pronounced in Wistar rats than other laboratory breeds of *R. norvegicus* such as Sprague–Dawley and Long–Evans rats^70^, ^71^ and a lack of other fear‐related behaviors such as freezing also were observed during exposure to TMT.^45^ Field studies on roof rats did not show effects of TMT on area avoidance or trap capture probability.^72^ Although there are no studies regarding bank vole avoidance of TMT, our results suggest a lack of response to this compound and could further indicate that responses to TMT may differ depending on the rodent strain or species. Additionally, intraguild predation^73^ also may explain this unexpected result. As a high predation on small mustelids by the red fox has been reported previously,^74^ we cannot discard the possibility that the smell of red fox feces also might indicate reduced presence of mustelids, thus reducing the probability of a bank vole meeting his major predators. However, to understand these potential ecological complexities explaining the attraction effects observed in our experiment, further field studies and further tests with wild‐caught rodents are needed.

Dose‐dependent behavioral responses of single compounds in rodents have been found by Jackson *et al*.^44^ in rats (*Rattus* spp.) where lower attraction rates were observed at higher concentrations, suggesting that odor saturation triggered fatigue in rats, leading to avoidance. In our study, odor saturation may not be the reason for the avoidance effects observed regardless of dose as our results with Heptanal and TMT indicate no avoidance. However, positive controls with nonpredator compounds to better exclude any odor saturation effect were not implemented in our study. Moreover, and in line with our results, Hansen *et al*.^75^ did not find the expected increase in repellent effects on common voles (*Microtus arvalis* Pallas, 1778) by higher doses of plant secondary metabolites in a laboratory study. This might indicate the need for further studies including a serial dilution method per compound in order to identify the bank vole's olfactory threshold.

Further studies also should address some of our experimental limitations. For example, a reliable indication of feeding inhibition or foraging can be done through the analysis of giving‐up densities by measuring the actual amount of food left over.^76^ Furthermore, habituation by rodents can diminish the efficiency of a repellent,^18^, ^24^ which was not tested in this study. Finally, sex differences in the response behavior may occur but owing to sample size, sex effects were not analyzed in our experiments.

## 5 CONCLUSIONS

In order to control plant damage, and seed removal and consumption during forest regeneration, repellents must be effective in both minimizing food contact or food manipulation (e.g. removing seeds from a target area) and inducing area avoidance. We demonstrate that 2‐PEA and 2‐PT both are effective as odor stimuli for triggering different avoidance‐related behaviors. Therefore, they may have the potential for future applications as repellents in both agriculture and forestry. However, field experiments are needed to determine their combined effects and dose‐dependence.

## CONFLICTS OF INTEREST

The authors declare no conflicts of interest.

## Supporting information


**Appendix S1**. Supporting Information

## Data Availability

The data that support the findings of this study are available from the corresponding author upon reasonable request.
