# Supplementary material for: Fear effects on bank voles (Rodentia: Arvicolinae): testing for repellent candidates from predator volatiles
Source: Pest Manag Sci. 2022 Jan 26;78(4):1677–85. doi: 10.1002/ps.6787 (PMC9306653; doi:10.1002/ps.6787)
Supplement: Supplementary file 1 — Appendix S1. Supporting Information [file PS-78-1677-s001.docx]

**Supplementary material**

**Methods for headspace collection from mink fur**

*Sample collection*

Fur samples of 10 female minks (*Neovison vison* Schreber) were obtained from the Center for Research in Mink Production, Health and Welfare (CPH Mink, University of Copenhaguen, Denmark) in June 2016. Between 1-5 grams of fur were trimmed from three dorsal areas of the animal. Animals were previously anesthetized with Ketaminol (50 mg/kg BW) and Xysol (10 mg/kg BW). Fur was stored at -20°C until the volatile extraction was performed (1-2 days after collection).

*Volatile extraction*

Dynamic headspace sampling was used to collect volatile compounds. Approximately 1.5 g of mink fur was placed in roasting bags (Toppits^®^, Minden, Germany). From inside of the roasting bag a PTFE aeration column (length: 60 mm; inner diameter: 3 mm, outer diameter: 4mm) containing 40 mg of the adsorbent polymer Porapak^®^Q (50/80 mesh, Waters Corp., Millford, MA, USA) was coupled to a rubber diaphragm vacuum pump (KNF Neuberger, Freiburg, Germany). Activated charcoal-filtered air was pumped through the column at a constant flow of 100 ml/min. Dynamic headspace sampling lasted 16 hours and the resulting extract was eluted with 500 µL pentane (*puriss p.a*.; Sigma-Aldrich, St. Louis, MO, USA).

*Chemical analysis*

All analyses were carried out at the Department of Plant Protection Biology, SLU (Swedish University of Agricultural Sciences, Alnarp, Sweden). We use a combined gas chromatograph 6890N GC and a mass spectrometer 5975 MS (GC-MS; Agilent Technologies Inc., Santa Monica, CA, USA) equipped with a HP-5 MS UI coated fused silica column (60 m x 0.25 mm; d_f_ = 0.25 µm, Agilent Technologies). The aeration extracts (2µL) were injected with a 7683B auto injector, splitless for 30 seconds at 230°C, followed by a 30 mL/min purge flow. Helium was used as the mobile phase with a constant flow of 35 cm/s. The initial oven temperature was set at 50°C (2 min hold) with an increase of 8°C/min to 225°C (10 min hold). The transfer line between GC and MS was set to 120°C. The mass spectral data was collected with an inert electron impact ion source at 70 eV. The scan range was programed from m/z 29 to 400 at 2 scans/s. The software MSD ChemStation (E.02.02.1431, Agilent Technologies) was used for the post-run analysis of the acquired GC-MS data. For the final identification of volatile compounds, we compared the retention times and mass spectra with a custom MS library, and those available from the NIST 14 and the Pherobase libraries. All samples were quantified by an external Kovats Index reference mix of octane through eicosane (C_8_ – C_20_) à 4ng/µL.

| **Table S.1.** Kovats' retention indices (RI) of volatile compounds from fur of 10 different minks. Retention time (RT) is given in minutes. RIs are means of samples where the compound was observed. *N* indicates how many samples the compound was found in. Reference RIs were obtained from the NIST or Pherobase online libraries. | | | | | |
| --- | --- | --- | --- | --- | --- |
| **CAS** | **Compound** | **RT (min)** | **RI** | **Reference RI** | ***N*** |
| 111-71-7 | Heptanal | 7.7 | 898 | 894^a^ | 10 |
| 80-56-8 | α-Pinene | 8.5 | 937 | 933^b^ | 7 |
| 111-66-0 | 1-Octene | 9.7 | 998 | 785^b^ | 1 |
| 3387-41-5 | Sabinene | 10.0 | 1013 | 964^a^ | 1 |
|  | Unknown | 10.0 |  |  |  |
| 3779-61-1 | *trans*-β- Ocimene | 10.0 | 1014 | 1027^a^ | 1 |
|  | Unknown | 10.0 |  |  | 3 |
|  | Unknown | 10.4 |  |  | 6 |
| 104-76-7 | 2-Ethyl-1-hexanol | 10.4 | 1032 | 1028^a^ | 1 |
|  | Unknown | 10.5 |  |  | 1 |
|  | Unknown | 11.8 |  |  | 1 |
|  | Unknown | 12.7 |  |  | 1 |
|  | Unknown | 13.7 |  |  | 1 |
| ^a^NIST library; ^b^Pherobase library | | | | | |

| **Table S.2.** Comparison of the probability of food contacts between the two arms of the Y-maze (treatment and control arm) during 10 odor puffs. Results were derived *a posteriori* from a generalized linear mixed model with a binomial error distribution using an interactions analysis with estimated marginal means (EMMEANS). Estimate values are back transformed to the response variable, so give differences between the probability of food contact in the treatment and control arms; positive values mean more interest in the treatment arm, and negative values mean more interest in the control arm. | | | | |
| --- | --- | --- | --- | --- |
| Compound | Estimate | SE | *z* ratio | *p* (>\|z\|) |
| 2-PEA | −0.32 | 0.06 | −5.52 | <0.01 |
| 2-PT | −0.16 | 0.06 | −2.97 | <0.01 |
| Indole | −0.10 | 0.06 | −1.77 | 0.08 |
| Heptanal | −0.02 | 0.05 | −0.31 | 0.76 |
| TMT | 0.21 | 0.05 | 3.48 | <0.01 |

| **Table S.3.** Relative time (in seconds) spent in the treatment and control arm detection zones of the Y-maze during 10 odor puffs. Results were derived *a posteriori* from a generalized linear mixed model with a Tweedie error distribution using an interaction analysis with estimated marginal means (EMMEANS). Estimates are back transformed to the response variable units (seconds), so give differences between the time spend in the treatment and control arms; positive values mean more interest in the treatment arm, and negative values mean more interest in the control arm. | | | | |
| --- | --- | --- | --- | --- |
| Compound | Estimate | SE | *t* ratio | *p* (>\|z\|) |
| 2-PEA | −240.90 | 128.60 | −1.90 | 0.03 |
| 2-PT | −150.00 | 93.90 | −1.60 | 0.11 |
| Indole | −162.50 | 110.60 | −1.50 | 0.14 |
| Heptanal | 13.60 | 53.70 | 0.25 | 0.80 |
| TMT | 188.7 | 102.00 | 1.85 | 0.04 |
